# An Outdoor Access Period Improves Chicken Cecal Microbiota and Potentially Increases Micronutrient Biosynthesis

**DOI:** 10.3389/fvets.2022.904522

**Published:** 2022-07-14

**Authors:** Lorena Varriale, Lorena Coretti, Ludovico Dipineto, Brian D. Green, Antonino Pace, Francesca Lembo, Lucia Francesca Menna, Alessandro Fioretti, Luca Borrelli

**Affiliations:** ^1^Department of Veterinary Medicine and Animal Productions, University of Naples Federico II, Naples, Italy; ^2^Department of Pharmacy, School of Medicine and Surgery, University of Naples Federico II, Naples, Italy; ^3^TaskForce on Microbiome Studies, University of Naples Federico II, Naples, Italy; ^4^The Institute for Global Food Security, Faculty of Medicine, Health and Life Sciences, Queen's University Belfast, Belfast, United Kingdom

**Keywords:** free-range chickens, caecal microbiota, environmental sustainability, mutual exclusion, zoonotic potential, gut microbiome prediction

## Abstract

Characterizing the gut microbiota of free-range and alternative poultry production systems provides information, which can be used to improve poultry welfare, performance, and environmental sustainability. Gut microbiota influence not only the health and metabolism of the host but also the presence of zoonotic agents contaminating food of animal origin. In this study, the composition and diversity of the cecal microbiota community of free-range grown chickens were characterized by 16S rDNA high-throughput Illumina sequencing. Significant differences were observed in the composition of chicken cecal microbiota at the time points of 28 days of age (Indoor group) and 56 days of age (Outdoor group), i.e., before and after the outdoor access period of chicken groups. The Outdoor group showed a richer and more complex microbial community, characterized by the onset of new phyla such as Deferribacterota and Synergistota, while the Indoor group showed an increase in Campylobacterota. At the species level, it is noteworthy that the occurrence of *Mucispirillum schaedleri* in Outdoor group is known to potentially stimulate mucus layer formation in the distal intestinal tract, thus being associated with a healthy gut. We also report a significant decrease in the Outdoor group of *Helicobacter pullorum*, highlighting that the lower abundance at the age of slaughter reduced the possibility to contaminate chickens' carcasses and, consequently, its zoonotic potential. As revealed by a mutual exclusion study in network analysis, *H. pullorum* was present only if *Bacteroides barnesiae*, an uncultured organism of the genus *Synergistes*, and *Bacteroides gallinaceum* were absent. Finally, microbiome predictive analysis revealed an increase of vitamins and micronutrient biosyntheses such as queuosine (Q) and its precursor pre Q0, in the Outdoor group, suggesting that the outdoor evolved microbiota of chickens do contribute to the vitamin pool of the gut and the biosynthesis of micronutrients involved in vital cell processes.

## Introduction

In recent decades, demand for free-range poultry meat has risen in Western societies, as a consequence of increasing consumer awareness of issues such as animal welfare, environmental impact, and food safety issues (1). Free-range rearing systems allow animals to express more natural behaviors and provide access to vegetation and other food sources. In broiler chickens, this is known to improve welfare (2) and potentially improve meat quality, composition, and organoleptic properties (3). Furthermore, free-range rearing can also influence production parameters such as body weight, feed intake, and feed conversion ratio (4). The gut microbiota composition of broilers is thought to be an important determinant of animal welfare, performance, and environmental impact (5). A healthy gut microbiome is essential not only to achieve optimal host performance but also to minimize the risk of zoonotic pathogens contaminating human food and adversely impacting consumer health (6). In general, greater microbial diversity is important to ensure host health status, because this plays a vital role in feed metabolism, immune system regulation, as well as the competitive exclusion of pathogenic organisms (7). Furthermore, several forage products are rich in functional molecules, some of which appear to be promising alternatives to antibiotics given that they contribute to the regulation of intestinal microbiota, enhance growth performance, and improve the immune response (8).

Many factors influence poultry gut microbiota diversity and composition, such as diet, rearing conditions, host genetics, and age (9–11). Age and breeding systems have been identified as key factors contributing to microbial diversity, but further studies are necessary to understand the importance of different rearing systems, particularly free-range systems (1). It will be important to establish a baseline for free-range systems to enable comparisons with factors such as diet and environment. Broiler chicken gut microbiota have been investigated for several years (12), but most efforts have focused on intensive systems of broiler production, and studies on other rearing systems are scarce. Therefore, the aim of this study was to characterize the composition, diversity, and predicted functions of the cecal microbiota community present in free-range broilers over their lifespan using next-generation sequencing (NGS) technologies. The intention was to broaden our understanding of the factors responsible for changing microbiota composition.

The present study examined a free-range system where broiler crossbreeds were reared in a certified brand breeding system in Southern Italy. This alternative system production is characterized by the use of GMO-free feed and the complete absence of antibiotics. It is also characterized by using renewable energy sources, a key point in the sustainability project of this rearing system. Finally, we report how symbiotic and commensal partnerships between bacteria and free-range broiler chicken could contribute to animal health and nutrition ranging from the metabolism of complex carbohydrates to the supply of important micronutrients such as queuosine and its precursors and vitamins.

## Materials and Methods

### Ethics Statement

All chickens were treated in accordance with the Directive of the European Parliament of the Council on the Protection of Animals Used for Scientific Purpose and in agreement with the Institutional Animal Care and Use Committee of the University of Naples Federico II, D.lgs n.26 04/03/2014. All experiments involving chickens were approved by the Bioethical Committee of the University of Naples Federico II, under the protocol number: 2018/0056762.

### Animals and Rearing Conditions

Hubbard broiler crossbreeds (ISA 956) were provided by a certified brand breeding located in a region of Southern Italy with a Mediterranean climate and a mean temperature of 21°C during the production period (February–March 2019). Chickens were reared for 56 days on a semi-extensive management system (from day 29, birds had free access to outdoor areas, and vegetation, seeds, fruits, soil particles, and insects became part of their diet) and slaughtered with an average body weight of 2 kg. Feed, provided *ad libitum*, consisted of 50–60% cereals and different proportions of wheat and soya according to age requirements (1–28 days 5% wheat and 30% soya, 29–56 days 11% wheat and 24% soya), supplemented with calcium carbonate, dicalcium phosphate, sodium chloride, and sodium bicarbonate. The vaccines administered were those against Newcastle disease, infectious bronchitis, and Gumboro disease. No antibiotics were used.

### Sample Collection

A total of 18 chickens were randomly selected from the same group at two time points, namely, 9 birds at 28 days of age (before having outdoor access: Indoor group) and other 9 birds at 56 days of age (before slaughter: Outdoor group), were euthanized by cervical dislocation and dissected under sterile condition. From each carcass, the ceca were tied at both ends, separated by sterile instruments from the rest of the gastrointestinal tract, placed in a sterile 15-ml Falcon tube, and stored at −80°C.

### Microbiota Sequencing and Data Analysis

Bacterial genomic DNA was extracted from ~0.18 g of cecal content using the QIAamp DNA Stool Mini Kit (Qiagen) according to the manufacturer's instruction. Sequencing samples were prepared according to the protocol 16S Metagenomic Sequencing Library Preparation for Illumina MiSeq System, as reported by Borrelli et al. (13). Demultiplexed paired-end reads from MiSeq (2 × 300 bp) were processed and analyzed using the Quantitative Insights into Microbial Ecology (QIIME2, version 2021.4) (14). Raw fastq reads were quality filtered to obtain amplicon sequence variants (ASV) using the DADA2 pipeline (15). Afterward, ASVs were classified within QIIME2 using the SILVA v138 database, with a classifier trained on the amplified regions (16).

To avoid sample size biases in subsequent analyses, a sequence rarefaction procedure was applied using a depth of 5,766 sequences/sample. Alpha (Shannon's diversity index and Chao1 index) (17) and beta diversity metrics (unweighted and weighted UniFrac distances) (18) were employed to study, respectively, the intra- and inter-group diversity of bacterial communities; group significance for alpha indices was calculated with QIIME2 plugins using the Kruskal–Wallis test; beta diversity was analyzed with permutational multivariate analysis of variance (PERMANOVA) performed in QIIME2 plugins, and PERMDISP was used to check for significant differences in dispersion (19, 20). Analysis of the composition of microbiomes (ANCOM) was used to identify the differential relative abundance of each bacterial phylum and species between groups (21). The linear discriminant analysis effect size (LEfSe) method (22) was used to identify taxa that differed in relative abundance between groups (LEfSe; *p* < 0.05 by the Kruskal–Wallis test, *p* < 0.05 by the pairwise Wilcoxon test and the logarithmic LDA score of 2.0). Microbial interactions were explored by generating Spearman's co-occurrence network based on the relative abundances of key species, using the CoNet plugin (23) for Cytoscape [3.9.0, (24)] by applying the following parameters: nonparametric Spearman's correlation coefficients with a minimal cut-off threshold of 0.6 (*p* < 0.05, Bonferroni corrected).

We inferred the microbial gene content from the taxa abundance using PICRUSt2 (25). To estimate the accuracy of PICRUSt2's prediction, the weighted Nearest Sequenced Taxon Index (NSTI) scores, which represent the phylogenetic distance for each ASV to its nearest sequenced reference bacterial genome, were calculated for each sample. The bacterial community samples had an average NSTI value of 0.18 ± SE 0.01, falling within a mid-range of reliability. Group differences in the inferred gene abundance of MetaCyc pathways (26) were identified by LEfSe (*p* < 0.05 by the Kruskal–Wallis test, *p* < 0.05 by the pairwise Wilcoxon test, and the logarithmic LDA score of 2.0) and summarized at MetaCyc class level in a Sankey diagram created using SankeyMATIC (https://sankeymatic.com).

## Results

### Chicken Health Status

At the end of their productive period, all birds were clinically healthy, and no illness signs, diarrhea, or mortality were pointed out. Necropsy showed neither signs of typhlitis nor macroscopic lesions in the gastrointestinal tract.

### Chicken Cecal Microbiota Sequencing and Overall Microbial Communities Landscape

Targeted sequencing of V3-V4 regions of 16S rRNA gene from cecal samples was performed to profile the gut microbiota structure and composition of free-range grown chickens throughout their whole productive period. After size filtering, quality control, and chimera removal, a total of 189,473 high-quality sequences were obtained from 18 cecal samples. After the rarefaction procedure, 5,766 reads/samples (refer to Supplementary Figures 1A,B showing rarefaction plots), annotated in 3,070 bacterial amplicon sequence variants (ASVs), 14 phyla, 156 genera, and 348 species were considered for subsequent analyses.

**Figure 1:**
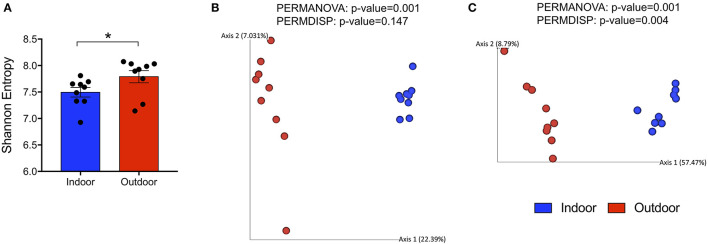
Global changes in structure and composition of cecal bacterial communities from indoor to outdoor rearing system. **(A)** Bar-dot plot showing Shannon Index as a measure of intra-group bacterial diversity (mean ± SEM; *p < 0.05). **(B,C)** Principal coordinate analysis (PCoA) plots based on unweighted and weighted UniFrac distances (5,766 sequences/sample), with the amount of variance along each axis in brackets. On the top of each PCoA plot are reported *p*-values of the PERMANOVA and PERMDISP tests used to estimate the significant differences in microbial community composition between the groups.

To evaluate alterations in the bacterial community structure between Indoor and Outdoor groups, we measured the microbial alpha diversity, using Chao1 (a richness estimator) and Shannon (accounting for both abundance and evenness of the taxa present) indices, and beta diversity analysis based on unweighted and weighted UniFrac distances. First, we observed that the outdoor access of the chickens from 28 days of age considerably increased the diversity and richness within microbial communities as shown by a Shannon Entropy increase in the Outdoor group compared with the Indoor group (Figure 1A). However, the Chao1 metric did not reveal differences in the number of species between the two groups (Supplementary Figure 1C). Moreover, microbiota harbored from the Outdoor group deviated from that of the Indoor group, specifically based on the presence/absence of phylogenetic clades as revealed by PERMANOVA analysis coupled with PERMDISP (*p* = 0.001 and *p* = 0.147, respectively) on unweighted UniFrac matrices (Figures 1B,C).

### Taxonomic Variation in Cecal Microbiota

**Figure 2:**
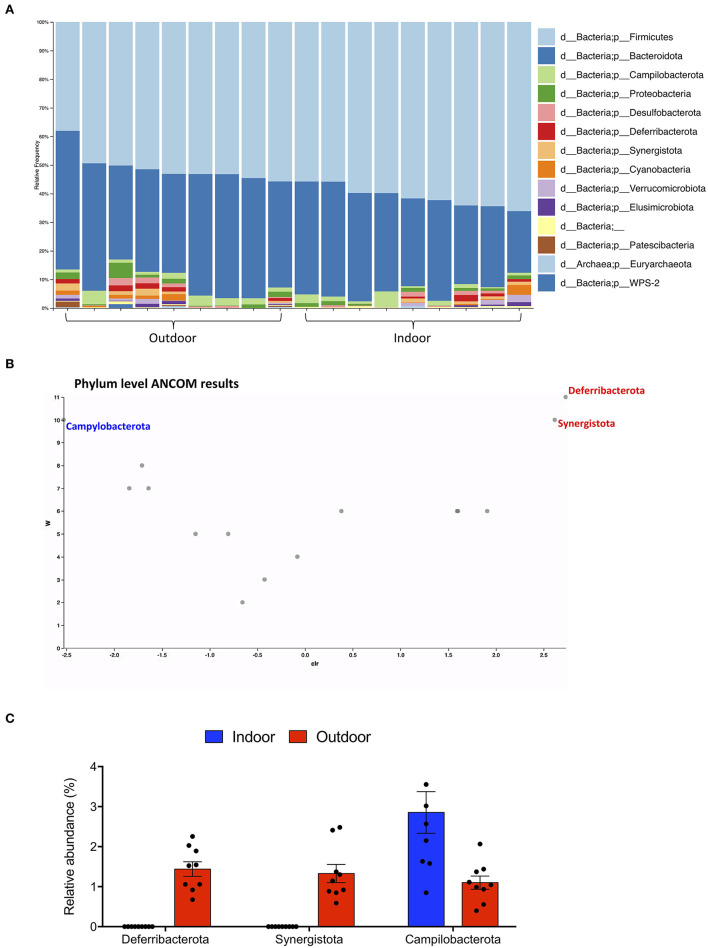
Phylum-level cecal microbiota assortment. **(A)** Bar chart showing the relative abundance of all bacterial ASVs taxonomically classified at phylum level in each sample. **(B)** ANCOM differential abundance volcano plot at the phylum level. Only significant bacterial phyla showing high w-stats are labeled and colored in blue or red for Indoor and Outdoor groups, respectively. **(C)** Bar-dot plot comparing the relative abundance of the phyla whose abundance was found to differ significantly between groups (mean ± SEM).

Gut microbiota composition was then studied at phylum and species taxonomic levels. A total of 7 and 14 different phyla were identified in the Indoor and Outdoor groups, respectively; Firmicutes and Bacteroidota were the dominant phyla in both the Indoor and Outdoor groups, representing more than 88% of the total communities (Figure 2A).

To obtain a clear picture of changes in gut microbiota between the two groups, we investigated the different abundant taxa at the phylum and species levels with the analysis of compositions of microbiomes (ANCOM). ANCOM identified as differently abundant the phyla Deferribacterota and Synergistota, which increased in the Outdoor group, and Campylobacterota, which increased in the Indoor group (Figures 2B,C).

**Figure 3:**
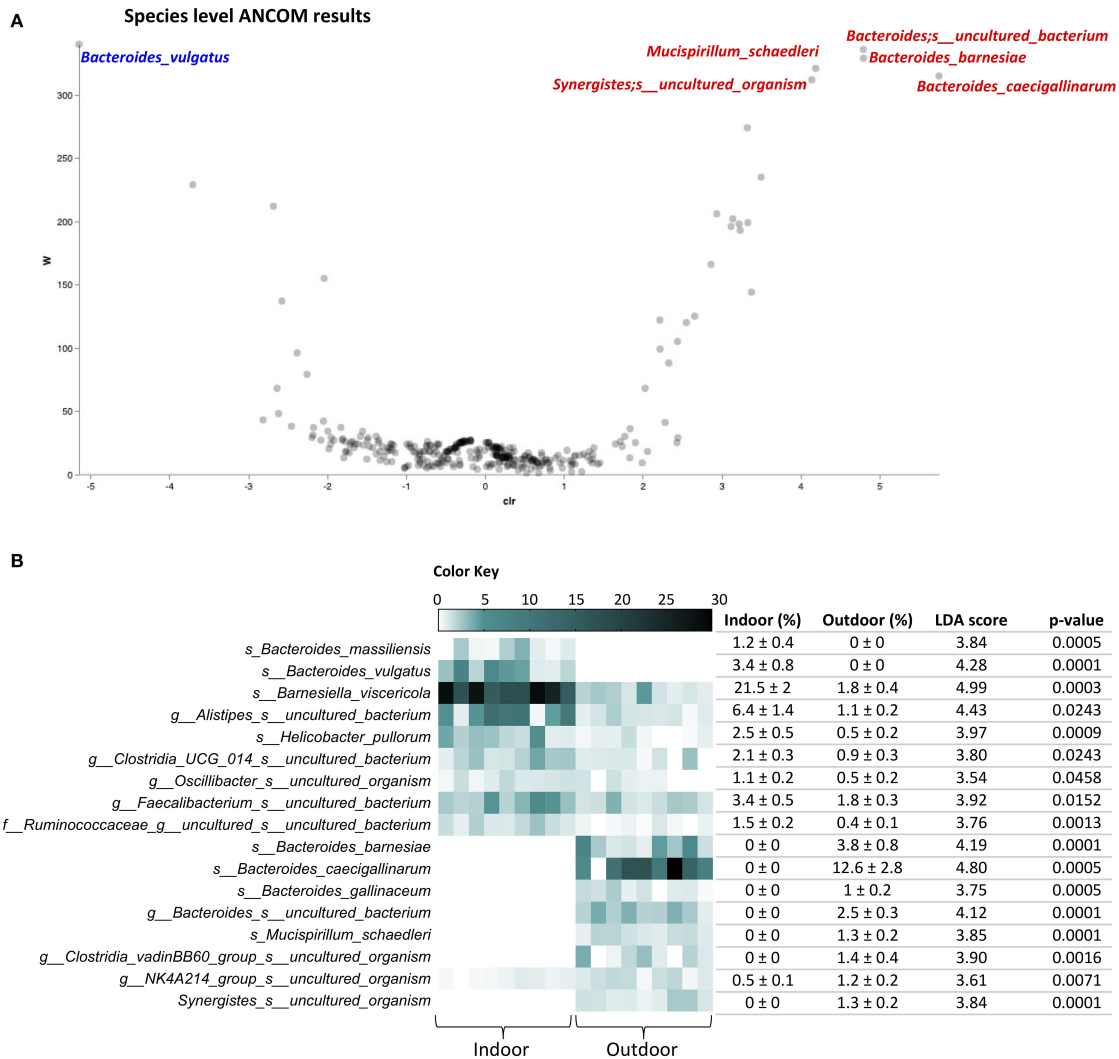
Species-level cecal microbiota assortment. **(A)** The ANCOM differential abundance volcano plot at the species level. Only significant bacterial species showing high w-stats are labeled and colored in blue or red for Indoor and Outdoor groups, respectively. **(B)** Bacterial species discriminating the cecal microbiota of Indoor and Outdoor chickens based on linear discriminant analysis (LDA) combined with effect size (LEfSe) algorithm (*p* > 0.05 for both Kruskal–Wallis and pairwise Wilcoxon tests and a cutoff value of LDA score above 2.0; only species with relative abundances >1% in at least one group are listed). Heatmap, mean ± SEM, LDA scores, and *p*-values are represented for each indoor-enriched and outdoor-enriched bacterial species, as identified by LEfSe.

Going in detail of the gut microbiota profiling, 118 and 151 different species with relative abundances >0.1% were identified in Indoor and Outdoor groups, respectively, reflecting the higher average species diversity based on an even abundance distribution of outdoor bacterial communities described by the Shannon index. A species-level analysis with ANCOM showed an increase of *Mucispirillum schaedleri, Bacteroides barnesiae, Bacteroides caecigallinarum*, and uncultured bacteria of the genera *Bacteroides* and *Synergistes*, together with a decrease of *Bacteroides vulgatus* in the Outdoor group (Figure 3A). Furthermore, ANCOM results were confirmed in a linear discriminant analysis of effect size (LEfSe) analysis, with the addition of several species with relative abundance >1%: an increase of *Bacteroides gallinaceum* and uncultured bacteria of the genera *Clostridia vadinBB60 group* and *NK4A214 group* (Oscillospiracae) with the outdoor access of the chickens; while *Helicobacter pullorum, Bacteroides massiliensis, Barnesiella viscericola*, and uncultured bacteria of the genera *Alistipes, Clostridia UCG 014, Oscillibacter, Faecalibacterium*, and family of Ruminococcaceae, marking the microbiota in the Indoor group (Figure 3B). Interestingly, *H. pullorum*, an enterohepatic *Helicobacter* species (EHS) recently recognized as an emerging human food-borne pathogen (27), showed a 78.8% decrease in the outdoor access of the chickens as obtained by calculating the mean percentage of *H. pullorum* reduction in the Outdoor chicken group with respect to Indoor chicken group.

Microbial co-occurrence and mutual exclusion network was created to explore the microbial interactions occurring among the identified bacterial species and to find potential mediators of the specific microbial assortment in response to the indoor or outdoor chicken time-point rearing system (Supplementary Figure 2). The network contained 14 nodes (microbial species) connected by 30 significant edges. *B. barnesiae* and an uncultured organism of genus *Synergistes* were the species with both the higher number of edges and closeness centrality scores, tending to co-occur with other 7 bacterial species and negatively correlate with *H. pullorum. H. pullorum* also showed mutual exclusion with *B. gallinaceum*, while it co-occurred with *B. vulgatus* (Supplementary Figure 2).

### Functional Prediction of Cecal Microbial Communities

**Figure 4:**
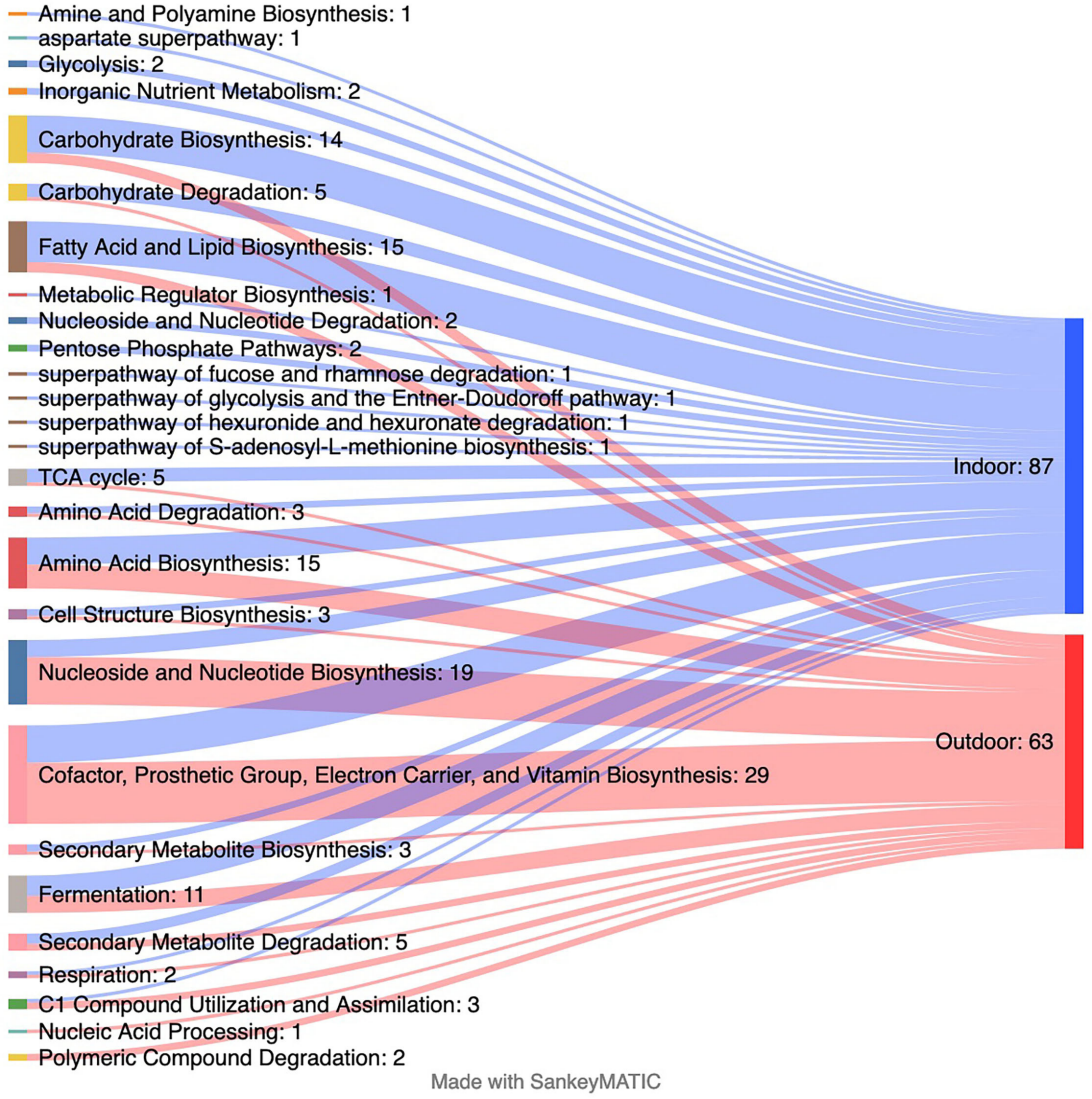
Changes in predicted metagenomics from indoor to outdoor rearing system. The Sankey plot displaying the number of predicted MetaCyc pathways collapsed at the MetaCyc class level that is significantly enriched in each group as assessed by linear discriminant analysis (LDA) combined with effect size (LEfSe) algorithm (*p* > 0.05 for both Kruskal–Wallis and pairwise Wilcoxon tests and a cutoff value of LDA score above 2.0). Refer to Supplementary Dataset 1 for the list of all significantly predicted functions at the MetaCyc pathway level.

Corresponding to the changes in several taxa, differences in 145 predicted genomic microbial pathways were found between the Indoor and Outdoor microbial communities (Supplementary Dataset 1; Figure 4). Functional metabolic pathways, clustered in MetaCyc classes of “adenosylcobalamin biosynthesis I (anaerobic),” “C1 Compound Utilization and Assimilation,” “Cofactor, Prosthetic Group, Electron Carrier, and Vitamin Biosynthesis,” “Nucleic Acid Processing,” “Nucleoside and Nucleotide Biosynthesis,” and “Photosynthesis and Respiration,” were significantly enriched in the Outdoor chickens compared with the Indoor chickens (Figure 4). Among these, the Outdoor microbial communities showed an increased count of pathways involved in vitamin/cofactor biosynthesis together with salvage of their precursor(s) from exogenous sources. In particular, biotin, riboflavin, cobalamin, queuosine (Q), preQ0 biosynthesis, and NAD and thiamin salvage (Supplementary Dataset 1) are augmented in free-range Outdoor chickens. Interestingly, several classes of pathways involved in the biosynthesis and degradation of macromolecules (such as fatty acid and lipid biosynthesis) were mainly enriched in Indoor chickens, suggesting that Outdoor chickens are sustained by the integration between microbiome-derived metabolites and the free-range environmental source.

## Discussion

This study provided a comprehensive sequence-based characterization of the cecal microbiota community of free-range grown chickens before and after their outdoor access period. Although microbiome analysis techniques have been applied to conventional poultry production systems (28, 29), there has been less focus on free-range systems. Reports on the cecal microbial community of broilers older than 42 days are scarce, and it is well recognized that many factors influence poultry gut microbiota composition and intestinal function such as diet, age, host genetics, breeding system, geographical location, and environmental conditions (1). Age-related processes occurring in the broiler lifespan such as feed changes, immune system development, and exposure to extraneous microorganisms result in gut microbiota shifts with advancing age. Studies have established that by day 21 of life, the microflora reaches a point of maturity; however, other production factors such as the beginning of the finisher diet from day 28 of life introduce further selective changes in the broiler gut microbiota, and by day 42 of life, over 200 genera colonize the intestinal tract (30). Therefore, it can be hypothesized that diet and rearing system are the principal variables that influenced the major changes in the cecal microbiota of the two groups. Noteworthy, the microbial community composition was considerably distinct before and after outdoor access, when different types of environmental resources, such as grasses, were introduced into the diet. This study agrees with other studies examining chicken cecal microbiota changes related to age and breeding system (31, 32). It finds that Firmicutes and Bacteroidota represented the dominant phyla of the cecal community regardless of whether they had outdoor access. 1 month after outdoor pasture, two new phyla emerged, namely, Deferribacterota and Synergistota. The changes in gut microbiota at the species taxonomic level, before and after outdoor access, were characterized by the onset of *Mucispirillum schaedleri, Bacteroides barnesiae, Bacteroides caecigallinarum*, and uncultured bacteria of genera *Bacteroides* and *Synergistes* after outdoor access of the chickens. Even if less is known about the role of these bacteria, evidence highlights their potential benefit for the host: bacteria from phylum Synergistota are mostly anaerobic microorganisms found in the animal digestive tracts and also in the soil (33); at present, the role of *B. caecigallinarum* has not been well and deep reported (34), but *B. caecigallinarum* represents an acetate and succinate producer (35), and it was demonstrated that it increased after the administration of fermented *Momordica charantia* juice, mitigating hyperglycemia, hyperinsulinemia, hyperlipidemia, and oxidative stress in diabetic rats (36).

Interestingly, the results obtained showed a clear difference in the assortment of Bacteroides genera between the two groups. The increase of *Bacteroides* genus in Outdoor microbiota is a potential positive finding due to its beneficial role in growth performance and the inhibition of *Clostridium perfringens* sporulation by its fermentation products (37), thus preventing the dramatic economic losses related to *C. perfringens*-associated necrotic enteritis (38).

Based on network analysis, *B. barnesiae* and an uncultured organism of the genus *Synergistes* might be proposed as keystone taxa whose presence drives the shift of composition and potential functioning of Outdoor microbiota. The network analysis also showed results of considerable interest regarding *H. pullorum*. Indeed, as revealed by the mutual exclusion pattern, *H. pullorum* was present only if *B. barnesiae*, an uncultured organism of the genus *Synergistes*, and *B. gallinaceum* were absent, suggesting these as key species driving the reduction of *H. pullorum*. In poultry, variable prevalence rates of this emerging food-borne pathogen have been reported from various regions with a range from 4 to 100% (27, 39); nevertheless, free-range and organic breedings have been poorly investigated so far. In our study, the significantly lower abundance of *H. pullorum* following the period of outdoor access (relative abundance <1% compared with >2% before) suggests that the potential for zoonotic infection (by means of broiler carcass contamination) is less likely. However, it's difficult to establish how the various factors involved in the changes of gut microbiota could have influenced our results, and further research is encouraged to deepen the unclarified aspects. We could hypothesize that the higher diversity in microbial community and the expansion of other taxa associated with a healthy gut could reduce the likelihood of colonization by this pathogen, and potential competitive exclusion strategies to limit the prevalence of *H. pullorum* might be investigated.

Among the minor phyla associated with a healthy gut, we have highlighted the presence of Deferribacterota and, at the species level, of *Mucispirillum schaedleri* following the outdoor access period. This observation is in line with other studies describing the microbial community of free-range chickens (6, 40). This bacterium is known to have a mucus-associated niche in the gut, and thus, it can be considered a marker for the health of the surface mucus layer in the distal intestinal tract (41). *Mucispirillum schaedleri* has also been reported to antagonize *Salmonella typhimurium* virulence and to protect mice against colitis by interfering with *S*. *typhimurium* invasion gene expression and competing for anaerobic electron acceptors (42).

Another very interesting finding of this study is that metagenomic pathway signatures before and after an outdoor access period differed in terms of their functional roles. Specifically, there were differences in a subset of functional roles (signature genes) that are required for *de novo* vitamin/cofactor biosynthesis. B vitamins are necessary cofactors for several aspects of human and animal metabolisms, including those concerning fat, carbohydrate, and DNA synthesis (43). Based on our findings, the biosynthesis of vitamins, such as B vitamins, also appears to be higher, underlining that the outdoor evolved microbiota of broilers contribute to the vitamin pool of the gut and that the host could benefit to some extent from the vitamin biosynthesis in the microbiota. Vitamins have remarkable antimicrobial activity *in vitro* as well as *in vivo*. Among the water-soluble vitamins, B1, B2, and vitamin B12, augmented after outdoor access in this study, were found to be effective, particularly against Gram-positive bacterial pathogens such as *Staphylococcus aureus, Staphylococcus epidermidis*, and MRSA, showing synergism with several antibiotics (44).

The gut microbiota provide a variety of micronutrients important for human and animal health. Among them, queuosine (Q), a vital micronutrient, synthesized exclusively by bacteria, represents a known and novel product arising from a microbe–host interaction. In bacteria, Q is synthesized *de novo*. On the contrary, eukaryotes lack the enzymes required for *de novo* synthesis of Q and hence rely on nutrient sources and additionally on their gut microbiome (45).

Gut microbiota represent a complex ecosystem that develops in close interaction with the host. In terms of Q metabolism, this environment is particularly complex, as specific microbes can be sources (46). Q plays a regulatory role in translation, cell proliferation, stress responses, and cell signaling. Q deficiency correlates with several phenomena and diseases including stress tolerance, cell proliferation, tumor growth, encephalomyelitis, and leukemia. Of importance, our metabarcoding study shows that the microbiota from broilers with a period of outdoor access are likely to have higher biosynthesis levels of Q and its Q precursor, pre Q0. This precursor is a key metabolite recruited in different pathways and used as a precursor of secondary metabolites, such as toyocamycin and sangivamycin, collectively referred to as deazapurines, in Actinomycetes. The nucleoside analog sangivamycin and its closely related nucleoside antibiotic toyocamycin showed a broad activity against arenaviruses, filoviruses, and orthopoxviruses; and an antitumor activity against leukemia and pancreatic cancer (47–49). This important finding suggests that gut microbiota evolved from the outdoor rearing with access to natural dietary sources increase the biosynthesis of Q and related metabolites. It will now be necessary to (i) undertake next-generation sequencing to fully understand the pathway changes, (ii) measure the differences in the concentrations of various Q pathway metabolites using techniques such as liquid chromatography mass spectrometry, and (iii) measure the extent of tRNA Qylation, in order to understand the importance of different rearing conditions.

This study provided new insights into the dynamics of microbial communities and predictions of metabolic functions in broiler chickens from a free-range system. Obviously, as known, these results coming from predictive imputed functions have evaluation limitations and should be interpreted cautiously. Therefore, further investigation, more replicates, microbiome, and metabolome studies are needed to better corroborate this perspective in a clearer view.

By targeting functional genes of chicken gut microbiota, new approaches might be explored to improve the growth performance and productivity of broiler chickens, and strategies promoting nutrient metabolism can be designed by introducing helpful bacteria possessing nutrient digesting genes involved in the maintenance of host health.

Diet and other environmental factors also represent powerful modulators of the gut microbiome, and further investigations are necessary to better understand how we can responsibly harness this inner ecosystem to make animals healthier, reduce antimicrobial use and dependence in livestock, and prevent antimicrobial resistance for an environmental health.

## Data Availability Statement

The original contributions presented in the study are included in the article/Supplementary Material, further inquiries can be directed to the corresponding author/s.

## Ethics Statement

The animal study was reviewed and approved by Institutional Animal Care and Use Committee of the University of Naples Federico II. Written informed consent was obtained from the owners for the participation of their animals in this study.

## Author Contributions

LB, LV, and LC contributed to the conception and design of the study, conducted the literature searches, wrote the manuscript, and performed the statistical analysis. LD, BG, and AP critically revised the manuscript. LB, FL, LM, and AF read, revised, and concurred with the final version of the review. All authors have made an intellectual contribution to the work and approved the submitted version.

## Conflict of Interest

The authors declare that the research was conducted in the absence of any commercial or financial relationships that could be construed as a potential conflict of interest.

## Publisher's Note

All claims expressed in this article are solely those of the authors and do not necessarily represent those of their affiliated organizations, or those of the publisher, the editors and the reviewers. Any product that may be evaluated in this article, or claim that may be made by its manufacturer, is not guaranteed or endorsed by the publisher.
