# Supplementary material for: An Outdoor Access Period Improves Chicken Cecal Microbiota and Potentially Increases Micronutrient Biosynthesis
Source: Front Vet Sci. 2022 Jul 14;9:904522. doi: 10.3389/fvets.2022.904522 (PMC9330014; doi:10.3389/fvets.2022.904522)
Supplement: Supplementary file 2 [file Table_2.DOCX]

Supplementary Material

# Supplementary Figures and Tables

## Supplementary Figures

**A**


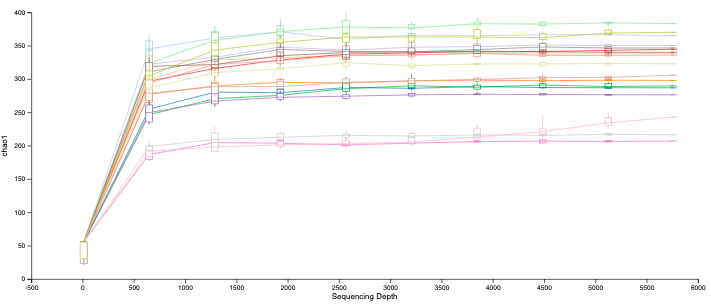


**B**


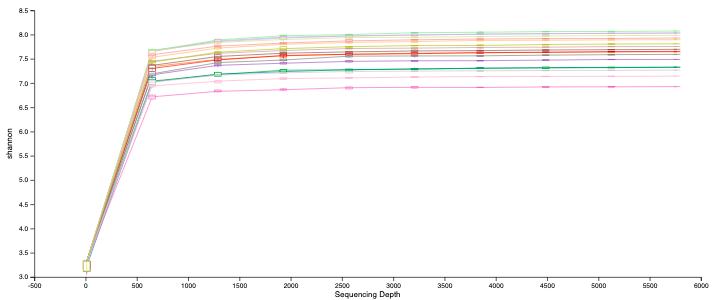


**C**

**Supplementary Figure 1.** A-B) ASV-level rarefaction curves based on Chao1 (A) and Shannon diversity index (B) of all samples. Rarefaction curves nearly reached a plateau, indicating that the sampling depth was sufficient to characterize the bacterial communities. C) Bar-Dot plot showing Chao1 metric (mean ± SEM).

**Supplementary Figure 2.** Co-occurrence network of species discriminating Indoor and Outdoor groups. In the networks: the nodes represent the species, and the edges show nonparametric Spearman correlations with a correlation coefficient > 0.6 (co-presence) or < -0.6 (mutual exclusion) statistically significant (p<0.05 after Bonferroni correction); green and red links denote a positive or a negative effect, respectively, where the direction of the arrow indicates the direction of the effect; label sizes are proportional to the closeness centrality score, feature used to measure the centrality of a node in the network; blue and red nodes represent their classification as Indoor or Outdoor key species, respectively.
